# Training and clinical testing of artificial intelligence derived right atrial cardiovascular magnetic resonance measurements

**DOI:** 10.1186/s12968-022-00855-3

**Published:** 2022-04-07

**Authors:** Faisal Alandejani, Samer Alabed, Pankaj Garg, Ze Ming Goh, Kavita Karunasaagarar, Michael Sharkey, Mahan Salehi, Ziad Aldabbagh, Krit Dwivedi, Michail Mamalakis, Pete Metherall, Johanna Uthoff, Chris Johns, Alexander Rothman, Robin Condliffe, Abdul Hameed, Athanasios Charalampoplous, Haiping Lu, Sven Plein, John P. Greenwood, Allan Lawrie, Jim M. Wild, Patrick J. H. de Koning, David G. Kiely, Rob Van Der Geest, Andrew J. Swift

**Affiliations:** 1 Department of Infection, Immunity and Cardiovascular Disease, University of Sheffield, Sheffield, UK; 2 INSIGNEO, Institute for In Silico Medicine, University of Sheffield, Sheffield, UK; 3 Norwich Medical School, University of East Anglia, Norwich, UK; 4 Radiology Department, Sheffield Teaching Hospitals NHS Foundation Trust, Sheffield, UK; 5 Department of Computer Science, University of Sheffield, Sheffield, UK; 6 Sheffield Pulmonary Vascular Disease Unit, Royal Hallamshire Hospital, Sheffield Teaching Hospitals NHS Foundation Trust, Sheffield, UK; 7 Multidisciplinary Cardiovascular Research Centre (MCRC) &, Biomedical Imaging Science Department, Leeds Institute of Cardiovascular and Metabolic Medicine, University of Leeds, Clarendon Way, Leeds, UK; 8 Division of Image Processing, Department of Radiology, Leiden University Medical Center, Leiden, Netherlands

**Keywords:** Right atrial area, Cardiovascular magnetic resonance, Convolutional neural networks, Artificial intelligence, Deep learning training, Clinical testing, Repeatability assessment, Mortality prediction

## Abstract

**Background:**

Right atrial (RA) area predicts mortality in patients with pulmonary hypertension, and is recommended by the European Society of Cardiology/European Respiratory Society pulmonary hypertension guidelines. The advent of deep learning may allow more reliable measurement of RA areas to improve clinical assessments. The aim of this study was to automate cardiovascular magnetic resonance (CMR) RA area measurements and evaluate the clinical utility by assessing repeatability, correlation with invasive haemodynamics and prognostic value.

**Methods:**

A deep learning RA area CMR contouring model was trained in a multicentre cohort of 365 patients with pulmonary hypertension, left ventricular pathology and healthy subjects. Inter-study repeatability (intraclass correlation coefficient (ICC)) and agreement of contours (DICE similarity coefficient (DSC)) were assessed in a prospective cohort (n = 36). Clinical testing and mortality prediction was performed in n = 400 patients that were not used in the training nor prospective cohort, and the correlation of automatic and manual RA measurements with invasive haemodynamics assessed in n = 212/400. Radiologist quality control (QC) was performed in the ASPIRE registry, n = 3795 patients. The primary QC observer evaluated all the segmentations and recorded them as satisfactory, suboptimal or failure. A second QC observer analysed a random subcohort to assess QC agreement (n = 1018).

**Results:**

All deep learning RA measurements showed higher interstudy repeatability (ICC 0.91 to 0.95) compared to manual RA measurements (1st observer ICC 0.82 to 0.88, 2nd observer ICC 0.88 to 0.91). DSC showed high agreement comparing automatic artificial intelligence and manual CMR readers. Maximal RA area mean and standard deviation (SD) DSC metric for observer 1 vs observer 2, automatic measurements vs observer 1 and automatic measurements vs observer 2 is 92.4 ± 3.5 cm^2^, 91.2 ± 4.5 cm^2^ and 93.2 ± 3.2 cm^2^, respectively. Minimal RA area mean and SD DSC metric for observer 1 vs observer 2, automatic measurements vs observer 1 and automatic measurements vs observer 2 was 89.8 ± 3.9 cm^2^, 87.0 ± 5.8 cm^2^ and 91.8 ± 4.8 cm^2^. Automatic RA area measurements all showed moderate correlation with invasive parameters (r = 0.45 to 0.66), manual (r = 0.36 to 0.57). Maximal RA area could accurately predict elevated mean RA pressure low and high-risk thresholds (area under the receiver operating characteristic curve artificial intelligence = 0.82/0.87 vs manual = 0.78/0.83), and predicted mortality similar to manual measurements, both p < 0.01. In the QC evaluation, artificial intelligence segmentations were suboptimal at 108/3795 and a low failure rate of 16/3795. In a subcohort (n = 1018), agreement by two QC observers was excellent, kappa 0.84.

**Conclusion:**

Automatic artificial intelligence CMR derived RA size and function are accurate, have excellent repeatability, moderate associations with invasive haemodynamics and predict mortality.

**Supplementary Information:**

The online version contains supplementary material available at 10.1186/s12968-022-00855-3.

## Introduction

Changes in the right atrium (RA) are important to recognise in the evaluation of patients with right ventricular (RV) failure [1–5]. Right atrial pressure (RAP) measured at right heart catheterisation is fundamental to the haemodynamic assessment of RV failure [6, 7] and predicts mortality in patients with pulmonary artery hypertension (PAH) [8, 9].

Accurate and repeatable measurements of cardiac chamber size and function are important for patient management [10]. A number of studies have revealed the prognostic significance of cardiovascular magnetic resonance (CMR) measurements in various cardiopulmonary diseases such as cardiomyopathies, pulmonary arterial hypertension (PAH), heart failure and ischaemic heart disease [11–15]. RA size and function measured by CMR can predict mortality [16–18] and the European Society of Cardiology (ESC) and European Respiratory Society (ERS) guidelines advocate the use of maximal (systolic) RA area for stratification of PAH patients [19].

RA measurements are often made manually on images viewed on patient archive and communication systems (PACS) or dedicated software packages with potential for observer variability. Image analysis tools differ between packages and the analysis does take a small but significant amount of time. With the advent of artificial intelligence (AI), deep learning using convolutional neural networks (CNNs), accurate cardiac chamber segmentations are possible [20–24]. Reference ranges for cardiac structure and function in healthy Caucasian adults from the UK Biobank population cohort were described for all four cardiac chambers using CMR [25]. Automated quality control (QC) in image segmentation was applied to the UK Biobank CMR study via the reverse classification accuracy (RCA) approach to categorize between successful and failed segmentations. This previous work showed that RCA has the potential for accurate and fully automatic segmentation QC on a per-case basis [26]. A deep learning based framework for automated, quality-controlled characterization of cardiac function from cine CMR has been established and reference values for cardiac function metrics were automatically derived from the UK Biobank cohort [27]. Fully automated CMR derived biventricular evaluation of function and morphology in a real-world setting has achieved good results without any operator interaction [28]. However, in the case of unseen anatomic variations, such as severe cardiac chamber shape changes and dilatation as in PAH, or significant artefact, then deep learning measurements may fail or be suboptimal [29].

Automation of RA area measurements may result in lower variability and assist clinicians to reach fast and robust clinical decisions. However, there are currently no studies that have automated CMR RA area metrics in the setting of PAH in which patients have varying degrees of RV failure, and the repeatability, correlation with invasive haemodynamics and success/failure rate in clinical populations remains unknown.

The aim of this study was to develop a quantitative CMR-based automated artificial intelligence (AI) analysis of the RA in a large cohort of patients with heart failure and PAH with varying aetiology and disease severity, and (i) determine the failure rate of the model in a large clinical registry, (ii) evaluate interstudy repeatability, (iii) directly compare the association of manual RA area and AI RA area with invasive haemodynamics and (iv) evaluate RA measurements as predictors of mortality.

## Methods

### Study population

A cohort of 365 subjects was used for training. This included a random selection of studies from 285 patients in the ASPIRE registry (several ASPIRE follow up scans were included with a total number of studies of 367). Sixty-six subjects from Leeds, including 29 healthy subjects and 37 patients with myocardial infarction of which 19 were acute and 18 were chronic. Fourteen healthy subjects from Leiden University Medical Centre (LUMC) were also included. The total number of studies included in the training cohort was 447. The demographics of the Leeds and Leiden subjects have been previously described [30, 31].

**Fig. 1:**
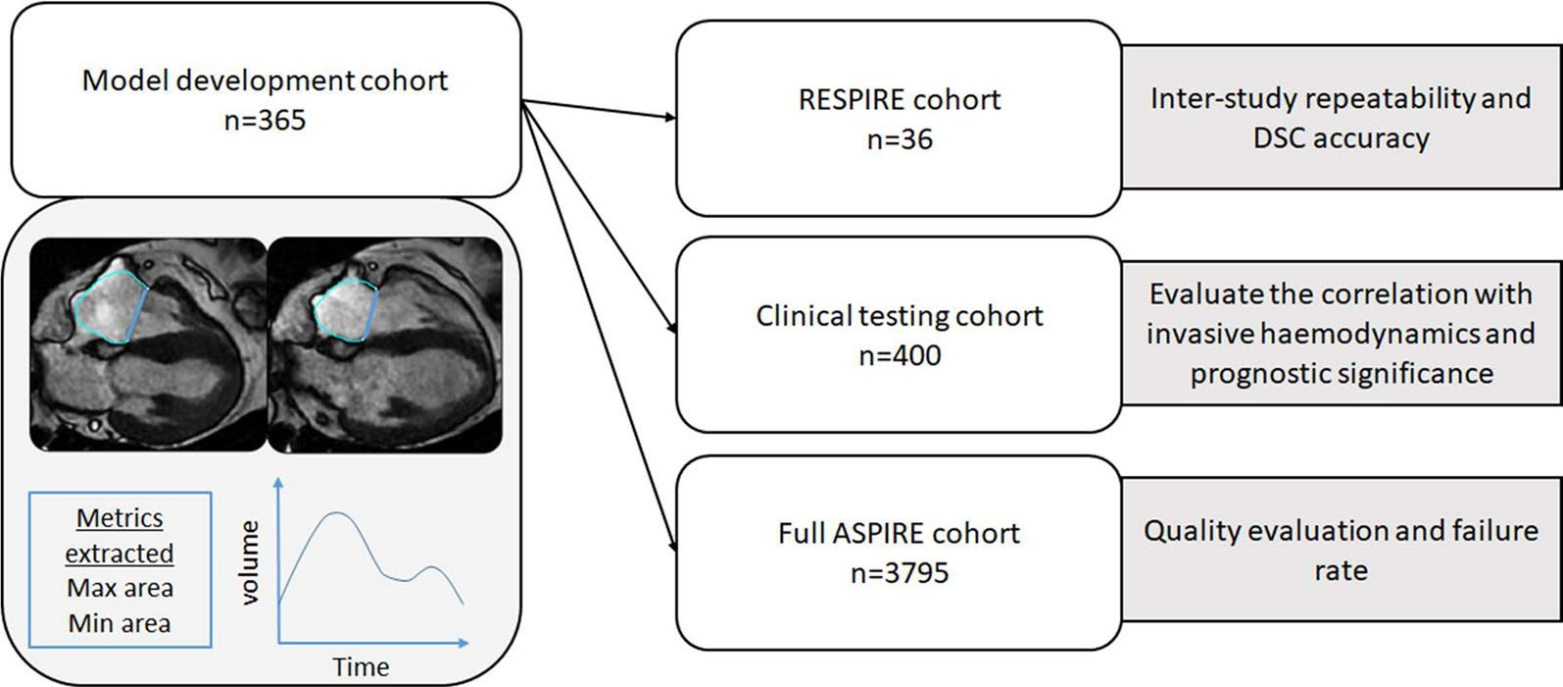
Study flow chart. Max = maximal; Min = minimal; DSC = DICE similarity coefficient

To test the model we used two populations. The first population included 36 patients CMR studies for prospective repeatability testing from the RESPIRE study (ClinicalTrials.gov Identifier: NCT03841344) [32]. The second population contained 400 patients CMR studies for clinical testing from the ASPIRE registry (ASPIRE, ref: c06/Q2308/8). For quality control and failure rate we included 3795 patients (5756 CMR studies, as follow up studies were included) from the ASPIRE registry (Fig. 1). Prospectively recruited patients provided written informed consent. Consent was waived for analysis of retrospective cases.

### CMR protocol

The training cohort included 1.5T (HDx, General Electric Healthcare, Chicago, Illinois, USA) and 1.5T (Ingenia, Philips Healthcare, Best, the Netherlands) studies. The testing cohort consisted of GE studies acquired in a clinical setting in the ASPIRE registry. The RESPIRE prospective cohort consisted of GE studies [32]. CMR studies in the testing cohort were performed using a whole-body scanner at 1.5T (HDx (General Electric Healthcare) [33]. Cine CMR acquisitions were made using a balanced steady state free precession (bSSFP) sequence. Following planning sequences, 4-chamber cine images were acquired. A stack of short axis images were acquired covering apex to base. Slice thickness and number of cardiac phases were 8 mm with 20 phases.

Leeds and Leiden CMR studies were performed on a 1.5 T system (Ingenia, Philips Healthcare) equipped with a 28-channel flexible torso coil and digitization of the CMR signal in the receiver coil. Vertical long-axis, horizontal long-axis, 3-chamber (left ventricular (LV) outflow tract-views), and the LV volume contiguous short axis stack cine imaging were defined using survey. All cines were acquired with a bSSFP, single-slice breath-hold sequence. Typical parameters for bSSFP cine were as follows: SENSE factor 2, flip angle 60°, TE 1.5 ms, TR 3 ms, field of view 320–420 mm according to patient size, slice thickness 8 mm and 30 phases per cardiac cycle.

### Image analysis

Four observers SA, FA, KK and AJS (with 2, 3, 13 and 11 years CMR experience, respectively) manually drew LV and RV and atrial contours in 4-chamber cine CMR views on all cardiac phases for the training and testing cohorts. All contours were drawn with observers blinded to the patient's clinical information. All manual contours were reviewed by an expert CMR reader (AJS). RV endocardial and epicardial surfaces were also manually traced from the stack of short-axis cine images to obtain RV volumetric and functional measurements as previously described [33]. MASS software (research version 2020; Leiden University Medical Center, Leiden, the Netherlands) was used for the manual contouring for developing the algorithm and repeatability testing).

### Deep learning training

CMR studies including a random selection of patients from the ASPIRE registry, subjects from Leeds, and from LUMC were used for deep learning training. The training process was performed in two stages. We trained two CNN models with different numbers of manually annotated 4-chamber view images in the training set. The validation set and test set used were the same for both of the CNN models. Since no hyper parameter tuning was performed in the current experiments a relatively small validation set of 6 subjects (180 images) was deemed sufficient to confirm model convergence during training and to confirm that the models did not suffer from overfitting. The test set consisting of 20 cases was used to compare the model performance of the initial model with the final model. Following this strategy we maximised the number of studies available for training. The initial model was trained on a combination of Philips (Leeds/LUMC, n = 80) and GE (Sheffield, n = 184) data (total n = 264). The contours used for training were all generated without the use of a CNN. For the final model 183 additional Sheffield GE scans were added. The contours for these additional cases were generated by reviewing and editing the contours generated using the base model. On average 50% of the contours generated by the initial model were manually edited for this set of cases. These cases were separate from the test cohorts.

The CNNs used for the experiments had an UNET-like architecture with 16 convolutional layers including residual learning units and was implemented using Python and TensorFlow. Input images were resampled to a fixed pixel spacing of 1 mm and cropped to a 256 × 256 image matrix size and zero filled when required. During training, data augmentation was performed on the fly by creating new training samples by randomly rotating, flipping, shifting and modifying image intensities of the original images. A total of 447 manually annotated 4-chamber cine series were used for training corresponding to 10,045 images. For training the Adam optimizer method was used, the learning rate was selected as 0.001 and cross-entropy was used as loss function. Each training batch included a random selection of 20 images. The number of epochs was set at a fixed number of 50, with all images used once in every epoch. The raw output of the CNNs is a labeled image, with the six possible label values corresponding to either one of the four cardiac cavities, the LV myocardium, or background. For each cardiac label, the largest connected component was extracted and a closed spatially smoothed contour around the extracted region generated. The area of the cardiac cavities was subsequently derived as the area surrounded by the generated contours. All experiments were executed on a standard PC with Intel Core i7 CPU with 64 GB of internal RAM memory equipped with an Nvidia GTX 1080 TI GPU with 12 GB of memory. The authors are happy to be contacted for research access to the Mass software and the AI segmentation tool upon request.

### Quality control

All automatically AI segmented RA area contours across all cardiac phases and resultant volume-time curves were evaluated by AS and scored as satisfactory, suboptimal or failure. In addition, the quality of the image acquisition was assessed for artefacts and slice position error. The definitions for QC were assigned prior to image review. Satisfactory was defined as either perfect contouring or minor errors that were not thought to affect the volumetric results. Suboptimal was defined as contours with errors deemed significant enough to affect the volumetric results. Failure defined as either absent contours or gross failure of the algorithm to segment the cardiac structures.

### Repeatability and agreement of the deep learning contours

To evaluate inter-study agreement two CMR scans were performed on the same day in two separate sittings as part of the RESPIRE study [32] for AI and manual measurements. In addition, interobserver agreement assessments, manual (AS) vs manual (FA), AI vs AS and AI versus FA were made. Agreement of the machine learning contouring model was evaluated by DSC. The DICE similarity for all cardiac cavities was computed in the 20 subjects in the test set. This was both for the baseline model as well as the final model.

### Association of manual and AI CMR measurements with invasive haemodynamics

Correlations with invasive haemodynamics were performed in patients in the ASPIRE registry clinical testing cohort who underwent right heart catheterisation within 48 h of CMR. The accuracy of RA CMR measurements to predict ESC/ERS mean RAP low and high-risk thresholds of 8 mmHg and 14 mmHg respectively, was assessed.

### Statistical analysis

Continuous variables are presented as proportions and means ± standard deviations. Normal distribution assessed by visual inspection of histograms and using the Shapiro–Wilk test. Variables that were not normally distributed were correlated using Spearman correlation coefficient. Univariate Cox regression Hazard ratios were calculated for AI and manual RA measurements to estimate the prognostic significance. Accuracy of RA measurements to predict RA thresholds performed using receiver operating characteristic analysis. Intraclass correlation coefficients and Bland–Altman plots were used to assess repeatability of manual and AI CMR metrics. Inter-rater reliability of the two observers grading of segmentation quality as satisfactory, suboptimal or failure was assessed using Cohen's kappa testing in a subcohort. Statistical analysis was carried out using SPSS (version 26, Statistical Package for the Social Sciences, International Business Machines, Inc., Armonk, New York, USA) and RStudio (version 1.2.5033, RStudio, Boston, Massachusetts, USA), and p value of 0.05 was considered statistically significant. For data presentation, GraphPad Prism (version 9.1.0, GraphPad Software, San Diego, California, USA) software was used.

## Results

### Patients

The ASPIRE registry in the training model included patients with left heart disease (15%), lung disease (12%), chronic thromboembolic PAH (21%), PAH (29%), other PAH (2%) and non-PAH (21%). The mean and standard deviation (SD) of the main haemodynamics of the ASPIRE registry in the training model is 10.4 ± 6.2 mmHg for mean RAP, 41.0 ± 15.5 mmHg for mean pulmonary arterial pressure, 13.4 ± 6.0 mmHg for pulmonary arterial wedge pressure, and 561 ± 466 dynes/m2 for pulmonary vascular resistance. The characteristics for the prospective repeatability, clinical testing and full cohort are presented in Table 1. In the clinical testing cohort, 218 of the 400 patients had died (54.5%) during a mean follow-up period of 1 year.

**Table 1:** Demographics, CMR and invasive haemodynamics of patients in the (i) RESPIRE (ii) Clinical testing and (iii) full cohort

	RESPIRE repeatability (*n* = *36*)	Clinical testing (*n* = *400*)	Full cohort (*n* = *3795*)
Demographics			
Age, yr	49.5 ± 15.9	55.4 ± 16.4	62.8 ± 15.3
Sex, F/M (F %)	30/6 (83)	283/117 (71)	2355/1440 (62)
BSA (m^2^)	1.9 ± 0.2	1.8 ± 0.2	1.8 ± 0.2
WHO FC I, *n* (%)	0 (0)	2 (1)	47 (1)
WHO FC II, *n* (%)	2 (6)	21 (5)	441 (12)
WHO FC III, *n* (%)	30 (83)	338 (85)	2743 (77)
WHO FC IV, *n* (%)	4 (11)	36 (9)	336 (10)
Diagnosis, *n* (%)			
Left Heart Disease	0 (0)	0 (0)	611 (16)
Lung Disease	0 (0)	0 (0)	632 (17)
CTEPH	0 (0)	0 (0)	728 (19)
PAH	36 (100)	400 (100)	1040 (28)
Other PAH	0 (0)	0 (0)	84 (2)
Other (not PAH)	0 (0)	0 (0)	677 (18)
Haemodynamics			
mRAP, mmHg	11 ± 7	10.4 ± 6.0	10.1 ± 6.0
mPAP, mmHg	52 ± 13	48.0 ± 13.7	40.8 ± 14.2
PAWP, mmHg	10 ± 3	10.3 ± 2.9	12.8 ± 5.9
Cardiac output L/min	4.5 ± 1.7	4.9 ± 1.5	4.9 ± 1.9
Cardiac index, L/min/m^2^	2.5 ± 0.9	2.8 ± 0.9	2.7 ± 1.0
PVR, dynes/m^2^	899 ± 512	720 ± 419	562 ± 419
MvO_2_, %	65.0 ± 9.1	63.5 ± 9.1	65.2 ± 9.3
CMR volumetric measurements			
RVESVI, ml/m^2^	25.4 ± 9.2	46.8 ± 28.2	37.3 ± 27.1
RVEDVI, ml/m^2^	63.3 ± 27.6	72.7 ± 35.5	62.6 ± 35.5
RVSVI, ml/m^2^	37.9 ± 20.7	25.9 ± 12.7	25.3 ± 15.4
RVEF, %	43.3 ± 10.0	39.1 ± 14.1	44.6 ± 16.1
CMR area measurements			
Automatic max RA area, cm^2^	22.6 ± 6.3	25.5 ± 9.8	25.8 ± 10.6
Manual max RA area, cm^2^	22.5 ± 6.3	26.0 ± 10.3	-
Automatic min RA area, cm^2^	15.0 ± 5.5	18.4 ± 9.4	18.5 ± 10.3
Manual min RA area, cm^2^	15.3 ± 5.7	19.3 ± 10.1	-

BSA, body surface area; CMR, cardiovascular magnetic resonance; CTEPH, chronic thromboembolic pulmonary hypertension; max, maximal; min, minimal; mRAP, mean right atrial pressure; mPAP, mean pulmonary arterial pressure; MvO2, mixed venous oxygen saturation; PAH, pulmonary arterial hypertension; PAWP, pulmonary arterial wedge pressure; PH, pulmonary hypertension; PVR, pulmonary vascular resistance; RHC, right heart catheterization; RVESVI, right ventricular end-systolic volume index; RVEDVI, right ventricular end-diastolic volume index; RVSVI, right ventricular stroke volume index; RVEF, right ventricular ejection fraction; RA, right atrial; WHO FC, World Health Organisation functional class. Data presented as mean ± standard deviation

### Quality control

Of 3795 patients (5756 studies) analysed by the AI model, 16 (0.3%) failed. 108 (1.9%) had suboptimal contours significant enough to be thought to affect the area measurements. In 72/108 patients, the 4-chamber slice was off-plane, with the most frequent error being inclusion of the LV outflow tract and suboptimal view of the RA. In 36/108 severe image artefact, typically breathing artefact or poor cardiac gating lead to suboptimal RA contours. In a randomly selected subcohort of 1018 studies, the scoring of satisfactory, suboptimal and failure showed excellent agreement between observer 1 and observer 2, with a high kappa statistic of 0.84.

### Segmentation agreement

**Fig. 2:**
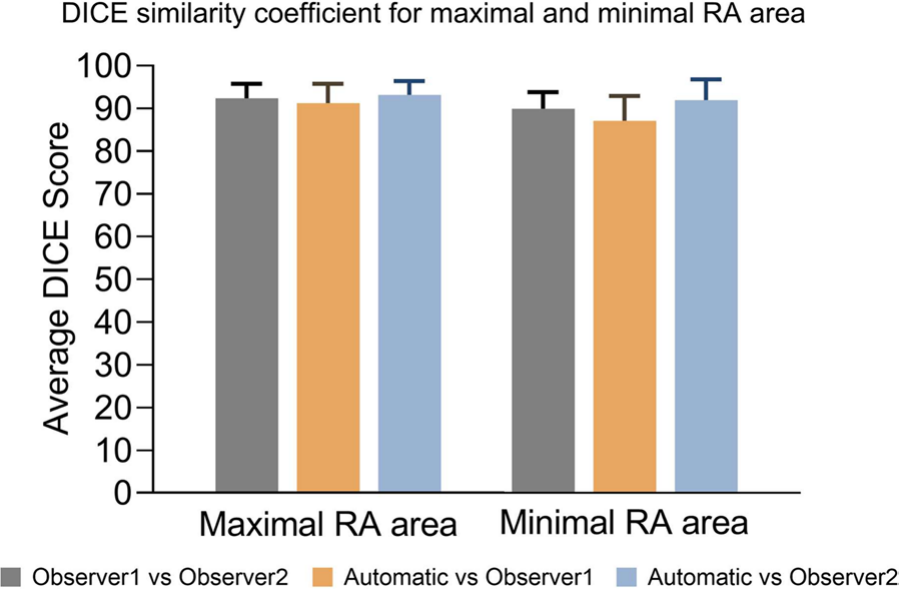
Right atrial **(**RA) measurements and DICE similarity coefficient. Maximal and minimal RA area DICE similarity coefficient results for (i) observer 1 vs observer 2 contour agreement, (ii) automatic vs observer 1 and (iii) automatic vs observer 2. RA = right atrial

Manual and automatic AI segmentation were assessed in the same day repeat studies from the prospective RESPIRE study. DSC showed high agreement (Fig. 2) comparing automatic AI and manual CMR readers, with a minimal bias towards either reader, validating similarity in the resulting contours. Manual contours made by observer 1 and observer 2 were closely related for both maximal RA area and minimal RA area. The mean and SD DSC metric for observer 1 vs observer 2, AI measurements vs observer 1 and AI measurements vs observer 2 is 92.4 ± 3.5, 91.2 ± 4.5 and 93.2 ± 3.2 for maximal RA area. The mean and SD DSC metric for observer 1 vs observer 2, AI measurements vs observer 1 and AI measurements vs Observer 2 is 89.8 ± 3.9, 87.0 ± 5.8 and 91.8 ± 4.8 for minimal RA area. The DSC for all four cardiac chambers before and after refinement for the 20 subjects in the test set are shown in Additional file 1: Table S1.

### Repeatability and agreement assessment

**Fig. 3:**
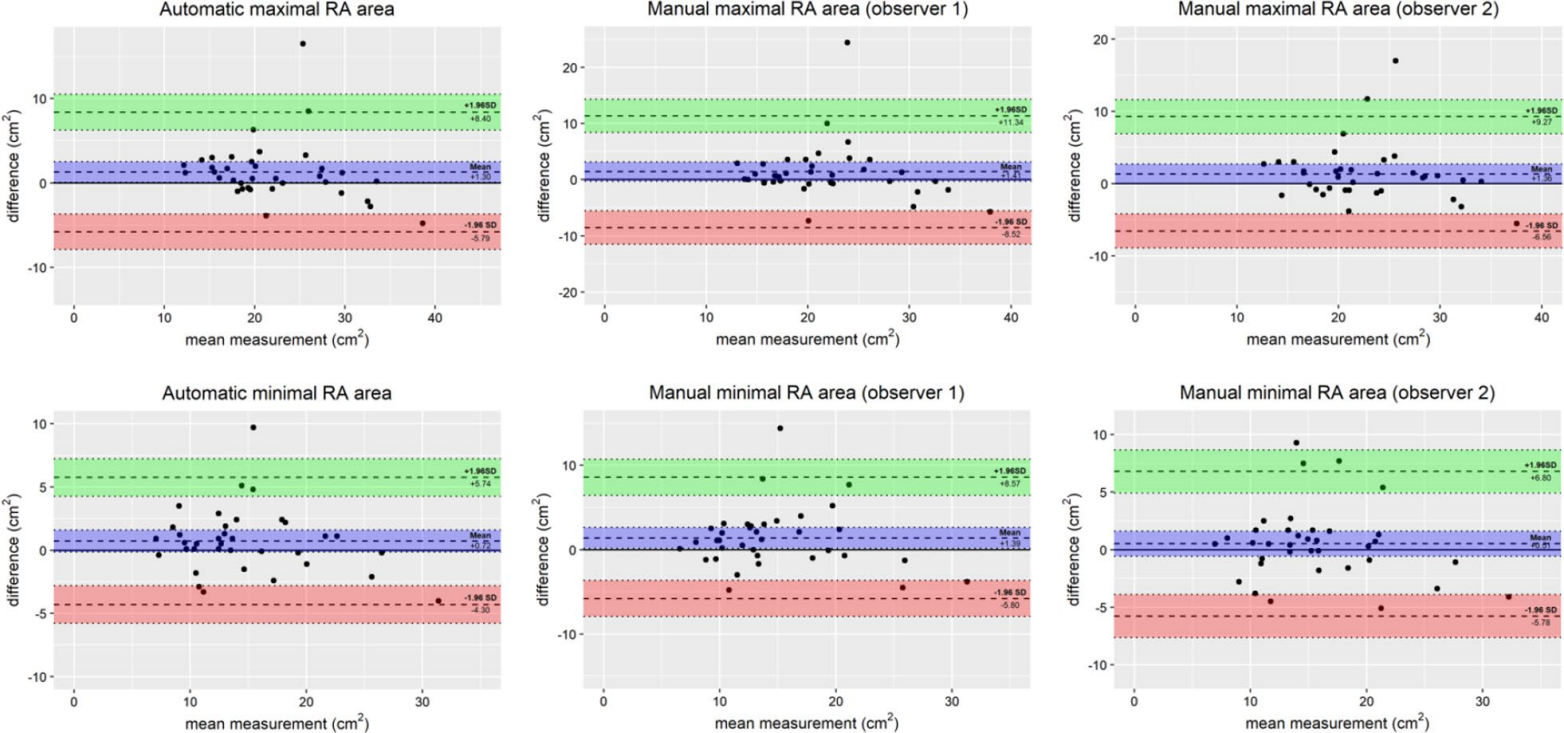
Bland–Altman plots and RA measurements. Bland–Altman plots showing CMR RA measurements scan-rescan results for (left) deep learning automatic AI measurements, (middle) observer 1 manual measurements, and (right) observer 2 manual measurements. CMR = cardiovascular magnetic resonance; AI = artificial intelligence; RA = right atrial

All AI RA measurements showed higher interstudy (scan-rescan) repeatability ICC 0.91 to 0.95, compared to manual measurements (observer 1 ICC 0.82 to 0.88, observer 2 ICC 0.88 to 0.91). Similar repeatability was also found comparing both observers with AI RA contours compared to observer 1 vs observer 2 ICC 0.96 to 0.98, see Tables 2, 3. Minimal bias was found for AI RA measurements, Fig. 3.

**Table 2:** Scan-rescan variability of automatic AI and manual right atrial CMR measurements

Interstudy (scan-rescan) variability (n = 36)						
	Automatic		Observer 1		Observer 2	
	ICC	95% CI	ICC	95% CI	ICC	95% CI
Max RA area	0.91	0.82, 0.96	0.82	0.65, 0.91	0.88	0.76, 0.94
Min RA area	0.95	0.89, 0.97	0.88	0.75, 0.94	0.91	0.84, 0.96

**Table 3:** Interobserver variability of automatic AI and manual right atrial CMR measurements

Interobserver variability (n = 36)						
	Automatic vs Observer 1		Automatic vs Observer 2		Observer 1 vs Observer 2	
	ICC	95% CI	ICC	95% CI	ICC	95% CI
Max RA area	0.99	0.97, 0.99	0.98	0.95, 0.99	0.98	0.94, 0.99
Min RA area	0.99	0.98, 0.99	0.97	0.92, 0.99	0.96	0.95, 0.99

AI, artificial intelligence; CMR, cardiovascular magnetic resonance; max, maximal; min, minimal; RA, right atrial

### Clinical testing cohort

In the clinical testing cohort (n = 400), RA area measurements made by AI and observers were comparable (Table 1). In the clinical testing cohort both manual and AI maximal RA area predicted overall all-cause mortality with similar predictive value, (hazard ratio 1.02 (95% confidence interval 1.01 to 1.03) and 1.02 (95% confidence interval 1.01 to 1.03) respectively, both p < 0.01). Manual and AI minimal RA area also showed a similar predicted mortality hazard ratio of 1.03 (95% confidence interval 1.01 to 1.02) and 1.02 (95% confidence interval 1.01 to 1.03), respectively, both p < 0.01.

Of the 400 patients identified for the clinical testing cohort, 212 patients underwent CMR and right heart catheterization (RHC) within 48 h. Moderate positive correlations were found between RA area measurements and mean RAP (mRAP) (AI, r = 0.64 and manual, r = 0.57). Moderate correlations of AI maximal RA area measurements with all invasive haemodynamics were found, see Table 4. The strongest correlation was found between minimal RA area and mRAP, r = 0.66), see Table 5.

**Table 4:** Pearson correlation (r) for the relation of manual maximal RA area and automatic AI maximal RA area with RHC parameters.  mRAP, mean right atrial pressure; PVR, pulmonary vascular resistance

RHC parameters	Manual maximal RA area (n = 212)		Automatic maximal RA area (n = 212)	
	r	*p*	r	*P*
mRAP	0.57	< 0.001	0.64	< 0.001
mPAP	0.38	< 0.001	0.46	< 0.001
Cardiac index	− 0.36	< 0.001	− 0.45	< 0.001
PVR	0.36	< 0.001	0.47	< 0.001
SvO2	− 0.41	< 0.001	− 0.48	< 0.001

**Table 5:** Pearson correlation (r) for the relation of manual minimal RA area and automatic AI minimal RA area with RHC parameters

RHC parameters	Manual minimal RA area (n = 212)		Automatic minimal RA area (n = 212)	
	r	*p*	r	*p*
mRAP	0.57	< 0.001	0.66	< 0.001
mPAP	0.40	< 0.001	0.50	< 0.001
Cardiac index	− 0.39	< 0.001	− 0.50	< 0.001
PVR	0.40	< 0.001	0.54	< 0.001
SvO2	− 0.44	< 0.001	− 0.55	< 0.001

RA, right atrial; AI, artificial intelligence; RHC, right heart catheterization; mRAP, mean right atrial pressure; mPAP, mean pulmonary arterial pressure; PVR, pulmonary vascular resistance; MvO2, mixed venous oxygen saturation

**Fig. 4:**
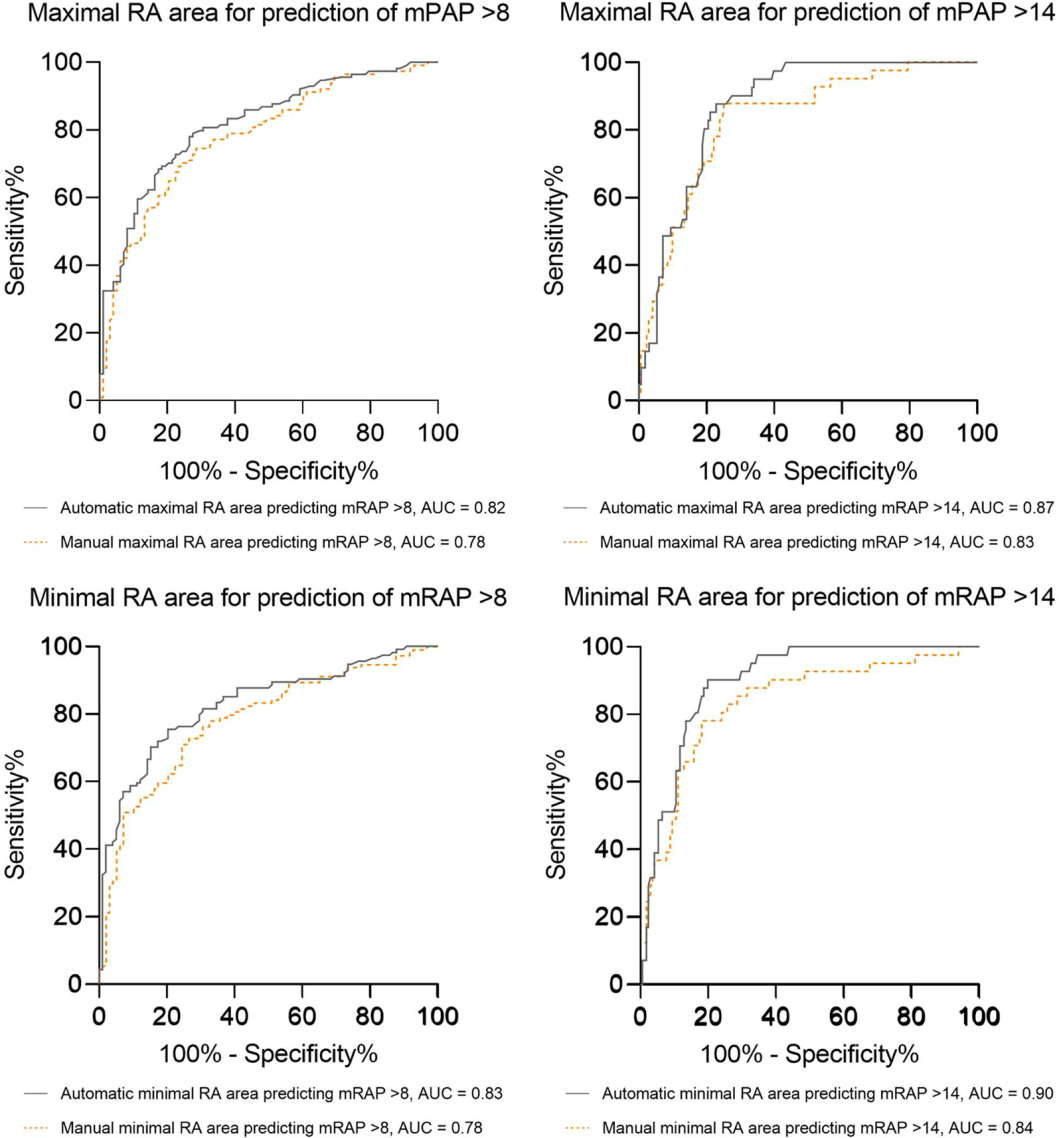
ROC curves and RA area measurements. ROC curves showing the accuracy of RA area measurements to predict mPAP at ESC/ERS guidelines risk thresholds. ROC = receiver operating characteristic; RA = right atrial; mPAP = mean pulmonary arterial pressure; ESC/ERS = European Society of Cardiology and European Respiratory Society; AUC = area under the curve

Maximal RA area could accurately predict mRAP low and high ESC/ERS risk thresholds (area under the receiver operating characteristic curve AI = 0.82 vs manual = 0.78 to identify low-risk patients with mRAP ≤ 8 mmHg and AI = 0.87 vs manual = 0.83 to identify high-risk patients with mRAP > 14 mmHg). Minimal RA area had a marginally highest accuracy for prediction of elevated mRAP, the strongest prediction was for mPAP > 14, area under the curve (AUC) 0.90, see Fig. 4. In comparison with manual measurements, automatic maximal RA area was not more accurate for detection of patients with mRAP > 8 mmHg and mRAP > 14 mmHg, (p = 0.11) and (p = 0.13), respectively. Automatic contouring of minimal RA area trended to suggest higher accuracy for predicting elevated mRAP > 8 mmHg and mRAP > 14 mmHg than manual measurements (p = 0.05) (p = 0.06), respectively, however these results are not of statistical significance.

## Discussion

This study shows that CMR RA area measurements can be fully automated using AI with a very low failure rate in a large clinical cohort with varying RA size and deformity. The variability of AI derived RA area measurements is lower than manual measurements in a scan-rescan cohort of patients with varying severities of RA size and function, and PAH. RA area measurements moderately correlate with invasive haemodynamics, and AI measurements can identify mRAP prognostic thresholds with more confidence than manual measurements, finally RA area measurements predict mortality with similar accuracy to manual measurements.

This study shows that fully automated Al-based contouring of the RA has a very low AI failure rate of ~ 2% in a large clinical population of patients with varying degrees of breathlessness, exercise limitation and aetiology of cardiac and pulmonary disease. The main reasons for failure were severe artefact, in particular poor cardiac gating, image noise and acquisition issues such as poor slice positioning of the 4-chamber slice, the latter the most common scenario. Such images cannot yield accurate RA area measurements by an observer or AI.

Using CMR, reference ranges for cardiac structure and function in healthy adults were previously described for all four cardiac chambers [25]. Automation of the QC process can potentially assist in validating AI algorithms. The potential for accurate and fully automatic segmentation QC has been demonstrated and applied to the UK Biobank CMR study using the RCA approach [26]. Reference values for cardiac function metrics were automatically derived from the UK Biobank and a deep learning based framework for automated, quality-controlled characterization of cardiac function from cine CMR has been confirmed [27]. Although, we advocate use of observer review in the QC process to maintain oversight of the segmented contours.

Assessment of interstudy (scan-rescan) repeatability is crucial to evaluate the utility of imaging measurements [34]. Interstudy repeatability is especially important for the comparison of automatic AI measurements with manual measurements [35]. We utilised a prospective scan-rescan study with rigorous study design [32] and show AI measurements are highly repeatable with marginally higher repeatability than manual measurements. Lower variability has advantages for more precise evaluation of changes in the RA following therapeutic intervention in trials and clinical practice, where treatment decisions are impacted by progressive structural and functional changes in the heart.

The ASPIRE registry includes a wide range of pathology including PAH, left heart failure, lung disease, chronic thromboembolic disease and patients found to have normal invasive haemodynamics. The AI 'seeing' a wider range of pathology is of paramount importance [20]. This is the first study to compare AI and manual measurements with invasive haemodynamic measurements of RAP. Here in this diverse population we identify a close correlation of AI RA area measurements with invasive mRAP, this combined with the low scan-rescan variability supports its potential use as a clinical tool. We show that RA area measurements are prognostic to a similar level as manual measurements. Further work to evaluate AI metrics in risk stratification is required as has been achieved for RV measurements [33]. In addition further work will be to clinically evaluate the range of physiological parameters that can be extracted from the AI segmentations, such as RA strain [36, 37] and potentially reservoir and conduit function [38, 39]. RHC measurements correlated strongly with AI RA measurements, indicating AI metrics may provide physiologically accurate measure of pathophysiological changes in the heart given their high consistency and repeatability.

## Limitations and future work

This is a single centre clinical testing of an AI algorithm developed in a multi-vendor multicentre cohort, with the clinical testing in the setting of a tertiary referral centre for patients with PAH. The imaging appearances and patient populations are likely representative of other PAH referral centres. The algorithm was generated in a multicentre setting, with single centre testing. Multicentre testing would be the next step to determine wider applicability of the algorithm. The current approach uses manual QC which is advantageous from a regulatory standpoint and maintains expert oversight of the AI. Future work to automate QC is of interest, however we consider manual review an important component of the system. Furthermore, future work will include evaluation of the utility of such automatic QC approaches in clinical populations.

This study developed an AI model for RA area estimation rather than volume. The rationale was to automate measurements made clinically and consistent with the ESC/ERS guidelines in PAH. Further work to develop and clinically evaluate a 3-dimensional or multislice RA volumetric model would be of value and work to extract physiological parameters previously suggested to be important [17] may be of benefit in future studies. Future work will be to explore the development of a four chamber AI prognostic model in PAH.

## Conclusion

In this study we have developed, tested and clinically validated an AI model to fully automate CMR RA area measurements. The data suggests great clinical applicability of AI derived RA measurements, in addition to time saving benefits.

## Supplementary Information

**Additional file 1: Table S1.** DSC values before and after refinement for all four cardiac chambers area.

## Data Availability

These can be provided upon request to the corresponding author.

## Abbreviations

AI: Artificial intelligence
AUC: Area under the curve
bSSFP: Balanced steady state free precession
CMR: Cardiovascular magnetic resonance
CNNs: Convolutional neural networks
DSC: DICE similarity coefficient
ESC/ERS: European Society of Cardiology and European Respiratory Society
ICC: Intraclass correlation coefficient
LUMC: Leiden University Medical Centre
LV: Left ventricle/left ventricular
mRAP: Mean right atrial pressure
PACS: Patient archive and communication systems
PAH: Pulmonary arterial hypertension
QC: Quality control
RA: Right atrium/right atrial
RAP: Right atrial pressure
RCA: Reverse classification accuracy
RHC: Right heart catheterization
RV: Right ventricle/right ventricular
SD: Standard deviation
