# Supplementary material for: Training and clinical testing of artificial intelligence derived right atrial cardiovascular magnetic resonance measurements
Source: J Cardiovasc Magn Reson. 2022 Apr 7;24:25. doi: 10.1186/s12968-022-00855-3 (PMC8988415; doi:10.1186/s12968-022-00855-3)
Supplement: Supplementary file 1 — Additional file 1: Table S1. DSC values before and after refinement for all four cardiac chambers area. [file 12968_2022_855_MOESM1_ESM.docx]

**Training and clinical testing** **of artificial intelligence derived right atrial CMR measurements**

Faisal Alandejani^1^, Samer Alabed^1,2^, Pankaj Garg^3^, Ze Ming Goh^1^, Kavita Karunasaagarar^4^, Michael Sharkey^1,4^, Mahan Salehi^1^, Ziad Aldabbagh^1^, Krit Dwivedi^1^, Michail Mamalakis^1^, Pete Metherall^4^, Johanna Uthoff^5^, Chris Johns^1^, Alexander Rothman^1,2,6^, Robin Condliffe^6^, Abdul Hameed^1,6^, Athanasios Charalampoplous^6^, Haiping Lu^2,5^, Sven Plein^7^, John P Greenwood^7^, Allan Lawrie^1^, Jim M Wild^1,2^, Patrick J.H. de Koning^8^, David G Kiely^1,2,6^, Rob Van Der Geest*^8^ and Andrew J Swift*^1,2^

^1^Department of Infection, Immunity and Cardiovascular Disease, University of Sheffield, Sheffield, UK

^2^INSIGNEO, Institute for In Silico Medicine, University of Sheffield, Sheffield, UK

^3^Norwich Medical School, University of East Anglia, Norwich, UK

^4^Radiology Department, Sheffield Teaching Hospitals NHS Foundation Trust, Sheffield, UK

^5^Department of Computer Science, University of Sheffield, Sheffield, UK

^6^Sheffield Pulmonary Vascular Disease Unit, Royal Hallamshire Hospital, Sheffield Teaching Hospitals NHS Foundation Trust, Sheffield, UK

^7^Multidisciplinary Cardiovascular Research Centre (MCRC) & Biomedical Imaging Science Department, Leeds Institute of Cardiovascular and Metabolic Medicine, University of Leeds, Clarendon Way, Leeds, UK

^8^Division of Image Processing, Department of Radiology, Leiden University Medical Center, Leiden, Netherlands

*Contributed equally to the manuscript

Corresponding author to: Dr Andrew J Swift, [a.j.swift@sheffield.ac.uk](mailto:a.j.swift@sheffield.ac.uk), Department of Infection, Immunity and Cardiovascular Disease, University of Sheffield, Sheffield, UK.

**Table S1** DSC values before and after refinement for all four cardiac chambers area

| **DICE** | **Baseline model (SD)** | | **Final model (SD)** | |
| --- | --- | --- | --- | --- |
| LV Endo ED | 93.93 | 2.58 | 94.37 | 2.31 |
| LV Endo ES | 89.71 | 4.26 | 90.47 | 3.61 |
| LV Epi ED | 93.78 | 2.52 | 95.12 | 1.44 |
| LV Epi ES | 92.53 | 3.50 | 93.26 | 2.37 |
| RV Endo ED | 91.68 | 3.66 | 94.30 | 2.33 |
| RV Endo ES | 88.26 | 4.59 | 91.35 | 3.09 |
| LA Endo ED | 91.05 | 4.45 | 92.04 | 3.56 |
| LA Endo ES | 86.87 | 7.75 | 90.03 | 4.67 |
| RA Endo ED | 92.63 | 5.84 | 93.56 | 3.26 |
| RA Endo ES | 92.37 | 4.82 | 93.10 | 3.50 |
| AVG | 91.28 | 4.40 | 92.76 | 3.01 |

*Definition of abbreviations*: DSC = DICE similarity coefficient; SD = standard deviation; LV = left ventricular; Endo = endocardial; ED = end-diastolic; ES = end-systolic; Epi = epicardial; RV = right ventricular; LA = left atrial; RA = right atrial; AVG = average.
